# HDL Function across the Lifespan: From Childhood, to Pregnancy, to Old Age

**DOI:** 10.3390/ijms242015305

**Published:** 2023-10-18

**Authors:** Brian V. Hong, Jingyuan Zheng, Angela M. Zivkovic

**Affiliations:** Department of Nutrition, University of California-Davis, Davis, CA 95616, USA; bvhong@ucdavis.edu (B.V.H.); jaczheng@ucdavis.edu (J.Z.)

**Keywords:** cardiovascular disease, children, cholesterol efflux capacity, elderly, high-density lipoproteins, lipids, pregnancy, neurodegenerative disease

## Abstract

The function of high-density lipoprotein (HDL) particles has emerged as a promising therapeutic target and the measurement of HDL function is a promising diagnostic across several disease states. The vast majority of research on HDL functional biology has focused on adult participants with underlying chronic diseases, whereas limited research has investigated the role of HDL in childhood, pregnancy, and old age. Yet, it is apparent that functional HDL is essential at all life stages for maintaining health. In this review, we discuss current data regarding the role of HDL during childhood, pregnancy and in the elderly, how disturbances in HDL may lead to adverse health outcomes, and knowledge gaps in the role of HDL across these life stages.

## 1. Introduction

High-density lipoprotein (HDL) particles are often recognized for their role in removing excess cholesterol from the body, known as reverse cholesterol transport [1]. However, in addition to, or as part of, this role in cholesterol clearance, HDL particles are critical during development [2] and have been implicated in several inflammation-related diseases including diabetes [3,4], chronic kidney disease [5,6], cardiovascular disease (CVD) [7,8], and Alzheimer’s disease (AD) [9,10]. The cholesterol content in HDL (HDL-C) has been well documented as an inverse marker of cardiovascular mortality across several prospective studies [11,12,13], and when measured earlier in life, is predictive of health status later in life. For example, low HDL-C measured during early adulthood (35–50 y) [14] and midlife (51–60 y) [14,15] is associated with increased AD risk after age 60, which suggests that monitoring, as well as interventions to improve HDL, should begin early in order to prevent disease decades later.

However, high HDL-C does not guarantee protection against overall health risk [16], and a U-shaped curve in the association between HDL-C and mortality risk has been observed, with HDL-C concentrations of both <30 mg/dL and >70 mg/dL associated with increased all-cause mortality risk [17]. The reasons for this complex relationship are not yet clear; however, it is clear that the simple measurement of HDL-C concentration does not provide information about HDL functionality, such as its ability to promote cholesterol efflux and its anti-inflammatory, antioxidative, and vasodilatory activities, among others [18]. The measurement of “dysfunctional” HDLs has emerged as an alternative therapeutic target over the measurement of HDL-C concentration [19]. For example, HDL’s ability to promote cholesterol efflux from macrophages is a more reliable predictor of CVD risk than HDL-C concentration [20,21,22], suggesting that the function of HDL is a promising approach for evaluating the complex role of HDL in health and disease.

The role of HDL in human health is largely studied during adulthood in participants with underlying chronic diseases; however, limited studies have examined the role of HDL in the health of children, during pregnancy, and in the elderly. This review highlights the current evidence on the role of HDL across the lifespan, particularly in children, during pregnancy, and in the elderly, and describes the limitations of current HDL research among these populations.

## 2. Children and Adolescents

### 2.1. HDL and Preterm Infants

Preterm birth is associated with cardiovascular events later in life [23], and is one of the main causes of neonatal death in the world [24]. Yet, early HDL-based therapies in preterm infants have not been fully explored. A long-term prospective study in preterm infants (*n* = 216) followed for 13–16 years into adolescence found improvement in both the low-density lipoprotein (LDL):HDL and apolipoprotein B (ApoB): Apolipoprotein A-1 (ApoA1) ratios in those infants who were breastfed compared to those who were fed preterm formula [25]. These findings suggest that breastfeeding early in life may influence lipoprotein profiles and cardiovascular health later in adulthood. Similarly, the benefit of breast milk on HDL extends to healthy infants. For example, infants born full term (*n* = 149) given exclusively breast milk for the first 6 months of life showed higher HDL-C:LDL cholesterol ratios compared to healthy infants (*n* = 150) on mixed feeding [26]. These findings reveal that HDL is responsive to diet early in life and that these early life exposures may have lasting impacts on lipoprotein metabolism into adolescence and adulthood.

HDL particle concentration and subclass distribution are additional parameters that have been found to be distinct in preterm infants. A small pilot study observed lower HDL particle concentration in preterm (29–34 weeks gestational age, *n* = 9) and very preterm (≤28 weeks gestational age, *n* = 6) infants at enrollment (0–5 days of age) compared to those at 2 weeks, with small HDL and small LDL particles being the predominant subclasses among the two groups [27]. Elevated small dense LDL particles have been linked to the development of CVD in children [28] and adults [29]. Dietary intervention in preterm infants (≤32 weeks gestational age) can ameliorate lipoprotein profiles, with early total parenteral nutrition from birth to 5 days of age increasing both plasma ApoA1 concentration and shifting HDL subclasses, as measured by gradient gel electrophoresis, from large HDL_2b_ towards smaller and more dense HDL_3c_ particles of similar value comparable to healthy term infants [30]. At 1 month of age, late-preterm infants (*n* = 25, 34–37 weeks gestational age) compared to term infants (*n* = 56) displayed lower cholesterol concentration within several HDL subclasses, including very large, large, medium, and very small HDL [31], which suggests a reduction in HDL maturation. This reduction in HDL maturation may be due to decreased lecithin-cholesterol acyltransferase (LCAT) activity. LCAT is an essential remodeling factor involved in HDL maturation [32] and is linked to CVD [33]. LCAT activity in cord blood [34] remained decreased in preterm infants during the first week of life compared to infants born full-term [35]. Furthermore, a pilot study comparing very-low birth weight preterm neonates (<32 weeks gestational age, *n* = 7) to full-term deliveries (*n* = 8) found decreased plasma ApoA1, an LCAT activator [36], in the HDL2 umbilical vein fraction of preterm babies by fast protein liquid chromatography isolation [37]. These studies suggest that a lack of LCAT activity and reduced ApoA1 concentration are factors that impair HDL maturation in preterm infants. Functional LCAT activity has been shown to be protective against CVD later in life [38]. In addition, HDL’s ability to efflux cholesterol, measured as cholesterol efflux capacity (CEC), is the initial step in the reverse cholesterol transport pathway and relies on LCAT for efficient cholesterol transport [20]. It is currently unknown whether and how preterm pathophysiology alters HDL CEC and other functions in infants. These limited data on the effects of preterm birth on HDL outcomes both during infancy and later in life provide tantalizing evidence that early life exposures may be critical in shaping life-long effects on HDL-mediated outcomes. Studies are needed to better understand how preterm birth alters pathways involved in HDL metabolism, and how these early life events shape future metabolic disease risk.

### 2.2. HDL in Children and Adolescents at Risk for Cardiovascular Events

CEC is one of many key functions of HDL that has consistently been shown to be directly linked with CVD [20,21,39]. Dyslipidemia mediated by disturbances in genes involved in cholesterol trafficking resulting in diminished HDL CEC in pediatric patients (3.6–5.1 y) makes these children susceptible to accelerated atherosclerosis compared to healthy children of similar age and sex [40]. In early childhood, HDL CEC was reported to be negatively associated with BMI at age 5, but not at age 9 [41], and was impaired in children with familial hypercholesterolemia [42]. Similarly, in adults HDL CEC was also found to be negatively associated with BMI and waist circumference [43], and in fact, this observation of a negative effect of obesity on HDL concentrations is so well documented in adults that low HDL-C is one of the 5 criteria for the diagnosis of metabolic syndrome [44]. In addition to the effects on HDL-C concentrations, obesity also has negative effects on HDL functional parameters. Obese children (11.8 ± 1.9 y) had impaired HDL function, including reduced HDL CEC and antioxidant capacities as well as LCAT activity [32]. Both dyslipidemic and normolipidemic overweight adolescents with a mean age of 13 years had reduced HDL CEC compared with normolipidemic normal-weight children of similar age and sex [45]. Together, these data suggest that disturbances in HDL metabolism and function can already be present in childhood and adolescence, and that some of the same factors that diminish HDL function and increase CVD risk in adults (e.g., obesity) are already present early on in life.

### 2.3. HDL in Children and Adolescents with Diabetes and Chronic Kidney Disease

Despite the fact that children with type 1 diabetes have normal HDL-C concentrations, loss of HDL function has been observed in this population [46,47,48]. Children (8–18 y) with type 1 diabetes had an impaired ability of HDL to exchange ApoA1 [46], a key process in the reverse cholesterol transport pathway that has been demonstrated to be associated with atherosclerosis [49] and positively correlated with ATP binding cassette transporter A1 (ABCA1)-mediated CEC, independent of HDL-C and ApoA1 levels [50]. In another study, children with type 1 diabetes and poor glycemic control had a lower proportion of large HDL_2b_ by native gel electrophoresis compared to age- and sex-matched non-diabetic children, despite a lack of difference in serum and HDL CEC [47]. By contrast, adults with type 1 diabetes were shown to have increased CEC and larger HDL particles measured by nuclear magnetic resonance (NMR) spectroscopy compared to non-diabetic adults, with no differences in HDL-C [51]. It is not clear whether these differences in HDL function and particle size in type 1 diabetic adults vs. children are due to underlying differences in biology or due to differences in the methods used to measure these HDL parameters [51]. Hyperglycemia is known to result in the glycation of ApoA1 and other HDL-associated proteins, which has been demonstrated to impair HDL function and affect HDL metabolism [52]. Thus, differences in glycemic control in both children and adults with type 1 diabetes may contribute to differences in HDL concentration, particle distribution and function.

Children with chronic kidney disease (9.8 ± 5.5 y) and end-stage renal disease (9.6 ± 5.0 y) also have dysfunctional HDL [53]. HDL of children with renal disease have an impaired ability to reduce monocyte adhesion to endothelial cells [53]; however, CEC was not affected in two different pediatric cohorts with chronic kidney disease (<16 y) [53,54]. Interestingly, this is similar to what has been observed for adult populations with kidney disease. Adult patients with end-stage renal disease also have impaired HDL functional parameters such as anti-oxidant capacity [55] but CEC may increase due to a preponderance of pre-beta HDL which effectively perform cholesterol efflux but which do not mature into the large, spherical HDL that carry additional functional proteins and thus perform additional functions [56]. It has been observed in adult patients with end-stage renal disease that this loss of HDL maturation is due in part to the loss of LCAT activity [57,58]. It is unknown whether similar mechanisms lead to loss of HDL maturation in pediatric kidney disease patients. Further studies in children are needed; however, the limited existing evidence already suggests that HDL disturbances in childhood kidney disease are likely to be similar to those observed in adults, confirming the importance of early detection and intervention.

### 2.4. Interventions That Improve HDL Concentrations and/or Function in Childhood and Adolescence

HDL functional biology during childhood and adolescence is poorly characterized. Nonetheless, interventions to improve child health measured through HDL markers have been investigated. Lipid-enriched supplements during pregnancy and postpartum in a malnourished population in Ghana can improve childrens’ HDL CEC levels at 18 months of age [59], with marked benefits on linear growth [60]. Interestingly, in these children, HDL CEC was associated with site-specific glycosylation of HDL-associated proteins [59]. Some of these associations were also observed in adults. For example, a positive correlation between alpha-1 antitrypsin (A1AT) glycosylation on amino acid position 70 (A1AT_70_5402 and A1AT_70_5412) and HDL CEC was observed in the children from Ghana as well as in healthy American adults [61]. These observations suggest that compositional parameters that are linked to HDL functionality in adulthood are already present in childhood, highlighting that similar markers can be used to assess the effects of interventions in children and adults.

In adolescents, interventions to mitigate atherogenic lipid profiles have been explored across several metrics of HDL function. Vertical sleeve gastrectomy in obese adolescent males (17.4 ± 1.6 y) enhanced both HDL CEC and anti-oxidative capacity, independent of both BMI and HDL-C concentrations at one year after the procedure [62]. Similarly, obese adults with type 2 diabetes undergoing sleeve gastrectomy showed improved ApoA1 exchange rate and ABCA1-independent CEC 5 years post procedure, though ABCA1-dependent CEC did not change [63]. In adolescents (14.9 ± 3.6 y), a 10-month lifestyle intervention program composed of dietary restriction, exercise, and psychological support improved both HDL CEC and antiatherogenic stimulation of endothelial nitric-oxide synthases [64]. Likewise in obese adult men, a one-year lifestyle modification consisting of aerobic activity and nutrition counseling improved both HDL-C and HDL CEC [65]. Together, these studies suggest that interventions to improve HDL in adults reflect similar changes to HDL in adolescents. Thus, therapeutic approaches and interventions that ameliorate HDL outcomes in adults may also be beneficial for adolescents. Given that chronic diseases like atherosclerotic CVD develop over the entire life course, interventions such as diet and exercise, which can be safely implemented over many decades and which have been shown to be effective in modifying HDL outcomes, are a desirable disease prevention strategy to implement early in life.

## 3. Pregnancy

### 3.1. Role of HDL during Pregnancy

HDL-C steadily increases during pregnancy and peaks between the 2nd and 3rd trimester [66,67], along with other lipid variables, including triglycerides, LDL cholesterol (LDL-C), and total cholesterol, which increase considerably throughout gestation [66]. Studies have shown that HDL-C can be positively associated with pregnancy outcomes; however, there is conflicting evidence on the extent to which HDL influences birth outcomes. On the one hand, HDL-C measured at 36 weeks gestation (26.5 ± 5.2 y, *n* = 320) was positively associated with the duration of pregnancy [68], and pregnant women (28.5 ± 4.6 y, *n* = 335) with a pre-pregnancy body mass index (BMI) ≥ 25 kg/m^2^ showed improved birth size when HDL-C increased by 10 mg/dL from preconception to 28 weeks gestation [69]. By contrast, others have found a negative association between maternal serum HDL-C and birth size measured during the second and third trimesters [70,71]. In a small cohort of healthy pregnant women (30.7 ± 3.96 y, *n* = 25), total HDL particle concentration measured by NMR during mid-gestation (22–28 weeks) was negatively associated with birth weight [72], which is in agreement with a case–control cohort (*n* = 5337) that found an association between higher total HDL particle concentration during mid-gestation (24–26 weeks) with small for gestation age infants at term, but no difference in HDL-C was observed [73]. An important consideration in these conflicting findings is that how HDL is measured heavily influences the results. HDL-C is a crude measure of the total amount of cholesterol transported as part of all HDL particles, which includes large and small HDL particles carrying different proportions of that cholesterol. However, there are significant differences between large and small HDL particles, despite the fact that the total size range is only about 5 nm. HDL particles that are 7 nm in diameter transport 10X fewer cholesterol molecules than HDL particles that are 12 nm in diameter [74]. There are also important differences in HDL particle composition by size. For example, apolipoprotein E (ApoE) and apolipoprotein J, which are both critically involved in AD, are enriched in large HDL, whereas A1AT, a protein known for its critical role in attenuating proteolytic damage during inflammation is found mainly on small HDL [75]. In addition to or as part of their ability to remove excess cholesterol from cells HDL confer anti-oxidant, anti-inflammatory, immuno-modulatory, anti-coagulant, anti-infectious, and many other functions, with as many as 16 functional subclasses dictated by protein composition [76]. Conditions where the pathology is a loss of HDL particles, such as that which has been observed in obesity and metabolic syndrome, can be measured by simple metrics such as HDL-C concentration, total amount of ApoA1, and total particle number. However, these simple measures may not capture the complexity of HDL, such as the relative proportions of different functional subclasses, which may be the critical differentiating factor in other conditions, such as in pregnancy.

### 3.2. HDL Subclasses during Pregnancy

Different subclasses of HDL transport different clusters of HDL proteins [75,76], and the presence of specific protein clusters can dictate HDL function [77]. A few studies have reported an increased proportion of larger HDL particles during pregnancy [78,79]. In a small cohort of healthy women at 9 weeks gestation, the authors observed a race-specific association with higher levels of large HDL measured by NMR among African American women with preterm birth (*n* = 12) versus African American women who gave birth at term (*n* = 14), but not in white women with preterm (*n* = 6) versus term (*n* = 17) births [80]; however, this observation needs further investigation in a larger cohort. In healthy Serbian women, large HDL_2a_ particles measured by NMR before delivery (37.2–37.3 weeks, *n* = 41) were shown to be negatively associated with birth length and head circumference [67]. In pregnant women from the Pregnancy, Infection, and Nutrition study (PIN) cohort (mean age 28 y, *n* = 715), composed of women with heterogenous pre-pregnancy weight (underweight to obese), a higher concentration of medium HDL by NMR during pregnancy at mid-gestation (24–28 weeks) but not at <20 weeks gestation was associated with an increased risk for preterm birth (<37 weeks), [81]. In a nested case–control study of mothers without severe chronic illnesses or preeclampsia (*n* = 323 cases, *n* = 671 controls) birthing children at term (≥37 weeks), NMR analysis during mid gestation (24–26 weeks) showed a higher proportion of small HDL and medium HDL as well as ApoA1 in mothers birthing small for gestational age infants compared to mothers with infants of normal birth weight [73]. However, the authors postulate that this observation was a consequence of placental dysfunction which may have increased maternal HDL levels, but future studies should define this relationship in detail. These studies suggest that the influence of HDL on birth outcomes differs substantially depending on the time of gestation at which HDL was measured. Furthermore, the changes to HDL during pregnancy suggest that modifications to HDL may also influence its function that may be linked to birth outcomes. The mechanisms of HDL metabolism leading to different HDL subclass distributions during pregnancy and its relationship with birth outcomes needs further investigation. A common limitation in these studies is the cross-sectional measurement of HDL that conceals the changes to HDL over the course of pregnancy as it relates to birth outcomes. Most cohorts were relatively healthy women, and, to our knowledge, it is not known yet whether alterations in specific HDL subclasses in chronic diseases (e.g., diabetes) during pregnancy are associated with birth outcomes. Other factors such as dietary intake before and during pregnancy were not reported, which may also influence HDL profiles.

### 3.3. HDL and Adverse Outcomes

Paraoxonase 1 (PON1) is an essential HDL-associated protein that has been linked to CVD and is protective against LDL oxidation through its antioxidant activity [82]. PON1 activity was found to be impaired in both obese adults and children [83], and its activity has been shown to be reduced in chronic renal failure [84], type 2 diabetes [85], and AD [86]. A longitudinal study in normal pregnant women (*n* = 50) found that PON1 activity decreases over the course of pregnancy which is accompanied by the natural increase in oxidative stress during pregnancy [87]. However, uncontrolled oxidative stress can be detrimental, where higher levels of oxidative stress during pregnancy play a role in the pathophysiology of preeclampsia [88], and increase the susceptibility of the fetus to develop CVD in adulthood [89]. Pregnant women with preeclampsia (29 ± 7 y, *n* = 19) during late pregnancy (35.2 ± 3.1 weeks) had reduced PON1 activity compared with healthy pregnant women of higher gestational age (38.4 ± 1.2 weeks) without preeclampsia (32 ± 3.8 y, *n* = 6), but no differences were observed in serum lipid levels including HDL-C [90]. The decrease in HDL PON1 activity without changes to HDL-C suggest that the function of HDL may be a more reliable predictor of pregnancy complications, similarly to the link between HDL function and CVD, which is also independent of HDL-C [20]. Likewise, in a cohort of white European women, women with preeclampsia (31 ± 6.5 y, *n* = 17) and their fetuses had lower ABCA1-mediated CEC compared with healthy normotensive pregnant women (28.2 ± 7.2 y, *n* = 17), but there were no differences in the concentration of maternal HDL-C between the two groups [91]. In a separate cohort, women with preeclampsia during pregnancy (31.4 ± 4.8, *n* = 42) had reduced ABCA1-mediated CEC at 6 months postpartum compared with postpartum women (32.4 ± 4.7 y, *n* = 44) who were normotensive during pregnancy, but, again, there were no differences in HDL-C concentration among the two groups [92]. The disturbance in HDL function during preeclampsia supports the importance of functional HDL on pregnancy outcomes. However, the limitations of these studies are the low number of participants, lack of follow up on mothers and their children, and uncertainty regarding the return of HDL function to normal values after delivery. Other analyses such as the association between maternal HDL function across pregnancy and birth outcomes (e.g., birth size) can be further explored. Furthermore, it is uncertain whether the changes in HDL function during pregnancy are causal for pregnancy complications. Many questions remain unanswered, yet current data point to important differences in HDL metabolism and function in healthy pregnancy vs. pregnancy with poor outcomes.

## 4. Elderly

### 4.1. HDL Cholesterol in Aging

Emerging evidence highlights the importance of HDL particles in aging biology. HDL-C concentrations have been found to be associated with age [93], physical activity [94], metabolic diseases [95], and cognitive decline [96]. While total HDL-C concentration has been a focus, specific impacts of aging on HDL metabolism, function, and composition remain understudied. This gap in knowledge warrants further investigation, as highlighted by the complex relationships observed between HDL-C, longevity, and cognition.

In elderly individuals, HDL-C is a strong predictor of longevity across several prospective studies [97,98,99]. Compared to healthy younger adults (41.0 ± 10.6 y, *n* = 200), octogenarians from Sicily (84.18 ± 3.6 y, *n* = 100) had significantly higher HDL-C concentrations [100]. Higher HDL-C concentration was also positively associated with better physical function (4 m walking speed, the short physical performance battery score, the basic and instrumental activities of daily living scales scores) in the octogenarian subjects (85.9 ± 4.9 y) of the ilSIRENTE study (*n* = 364) [97]. A genome wide association study examining relationships between the age at death of parents of middle-aged UK Biobank participants of European decent (*n* = 75,244) showed that longevity was associated with HDL-C concentration only in the parent group with extreme longevity (father ≥ 95 y, mother ≥ 98 y, *n* = 1339) [101]. In a similar study, researchers recruited 312 offspring from longevity historical families and 298 controls from non-longevity historical families in the Bama Aging Study cohort, aiming to understand the potential relationships between HDL-C, apolipoprotein E (*APOE*) genotype, and longevity [98]. HDL-C was significantly higher in the longevity group, while *APOE* genotype was not associated with either HDL-C or age [98].

In a study of 139 Ashkenazi Jewish centenarians (age 95–107 y), plasma HDL-C concentrations were significantly and directly correlated to Mini-Mental State Examination (MMSE) scores [102]. When compared within the centenarians, female, but not male, subjects with lower MMSE scores (<25) had significantly lower plasma HDL-C compared to those with higher MMSE scores (25–30) [102]. Compared to female nonagenarians (92 ± 4.0 y, *n* = 280), male nonagenarians (91 ± 1.0 y, *n* = 107) had significantly lower HDL-C concentrations, which were associated with MMSE scores only in the male subjects after adjustment for covariates [103]. In the Baltimore Longitudinal Study of Aging, high LDL:HDL ratio was associated with an increased risk of developing AD 5 to 7 years before diagnosis [104]. However, low HDL-C (<50 mg/dL) alone did not show significantly higher relative risk for developing AD compared to normal HDL-C concentration [104]. Together these data suggest a relationship between high HDL-C concentrations and protection from cognitive decline in elderly individuals, and particularly the oldest old.

However, HDL-C concentrations have also been found to be higher in populations with cognitive decline. In a study investigating the relationship between cognitive decline, fat-soluble vitamins, and *APOE* genotype in aging adults and elderly (65.31 ± 6.30 y), HDL-C was higher in the mild cognitive impairment (MCI) group (*n* = 583) compared to the normal group (*n* = 1171), though this comparison was not adjusted for any genotype effects [105]. Higher HDL-C was found in the cognitively normal group with *APOE*2 genotype, and in the MCI group with *APOE*2 and *APOE*4 genotype, compared to those with the *APOE*3 genotypes [105]. HDL functional capacity was recently found to be differential by *APOE* genotype in elderly individuals, with *APOE4* carriers having lower CEC and LCAT activity regardless of dementia diagnosis [106]. *APOE* genotype is also well known to be associated with HDL-C concentrations, LDL-C and total cholesterol concentrations [107], as well as CVD risk and mortality [108]. Thus, in studying the relationships between HDL and aging outcomes, it is important to account for *APOE* genotype, especially in the elderly, where individuals who have not already died from heart disease will be disproportionately overrepresented.

HDL-C status in elderly was also shown to be differentiated by eating patterns. In a study group of community-dwelling elders in Northern China with normal HDL-C (67.3 ± 5.9 y, *n* = 2646) and low HDL-C (67.3 ± 5.9 y, *n* = 741) levels, participants with the highest scores for a balanced diet had a decreased risk for developing low HDL-C, characterized as having <1.04 mmol/L (40 mg/dL) in the group with a BMI of 27.1 kg/m^2^ or above (overweight to over obese) [109]. Conversely, there was a significant negative association between those with a high intake of the Western diet and low HDL-C in the group with a BMI of 21.6 to 24.8 kg/m^2^ (normal to slight overweight). Interestingly, a higher score for the thrifty dietary pattern was also associated with increased risk of low HDL-C, especially in the group with a BMI of 21.6 kg/m^2^ and below (normal to underweight). These observational data suggest that diet quality is linked with HDL-C status and that the effects of diet on HDL are mediated by BMI status, where both low and high BMI can affect HDL-C concentrations. Together, the data on HDL-C in aging indicate that HDL-C concentrations are associated with differential outcomes in aging, that *APOE* genotype is an important mediator in the relationship between HDL-C and aging related outcomes, and that dietary patterns influence HDL-C in elderly individuals.

While some studies show higher HDL-C levels are associated with longevity and cognitive function in the elderly, conflicting findings emerge linking HDL-C to cognitive outcomes. For example, higher HDL-C correlates with better cognition in centenarians [102], yet also occurs in MCI versus normal aging [105]. Reasons for these inconsistent relationships remain unclear across life stages including aging, pregnancy, and birth outcomes [68,70,71], but may involve factors such as *APOE* genotype that impact links between HDL and cognition. Additionally, diet quality impacts age-related HDL-C changes, highlighting the role of modifiable lifestyle factors [109]. Overall, total HDL-C concentration imperfectly predicts cognition and longevity, underscoring limitations as a surrogate marker in the elderly. A deeper understanding of HDL metabolism in the context of aging is needed to elucidate these discrepancies and better assess HDL’s multifaceted roles across the lifespan.

### 4.2. HDL Functions in Aging: Cholesterol Efflux and Aβ Carrying Capacity

Few studies have examined key functional metrics such as HDL CEC and amyloid β (Aβ) carriage in elderly. To fully understand the role of HDL, measurement of HDL functionality is crucial in addition to measurement of HDL size, number, and concentrations, yet these metrics are lacking in aging research.

In a small study, compared to young subjects (20–30 y, *n* = 8), elderly individuals (65–70 y, *n* = 9) presented lower levels of ABCA1-mediated CEC, specifically for the HDL_3_ subclass [110]. This reduction in efflux capacity may have been due to higher levels of damage to the HDL particles since the HDL of elderly subjects had higher amounts of ApoA1 modification (i.e., oxidation) [110]. In a larger study investigating the effects of extra virgin olive oil (EVOO) consumption on HDL CEC, elderly subjects (70.72 ± 5.6 y, *n* = 57) had lower HDL CEC than young subjects (31.81 ± 6.79 y, *n* = 27), as well as higher small HDL and lower large HDL at baseline [111]. The study also showed that 12-week consumption of EVOO at 25 mL/day improved HDL CEC of the elderly subjects to the same level comparable to young subjects at baseline, though HDL CEC for the young subjects also improved and was significantly higher than that of the elderly subjects at the end of the study [111].

In an early study on the role of cerebrospinal fluid HDL on the development of AD, the HDL_1_ subclass was found to have increased soluble amyloid β (sAβ) and increased apolipoprotein in the AD group compared to age-matched aging normal human subjects, and that sAβ molecules were associated with ApoE and apolipoprotein J in the HDL_2_ and HDL_3_ subclasses [112]. HDL levels were shown to positively associate with serum neurofilament light levels, a biomarker for neuro-axonal damage, in study subjects age >60 years, but not in subjects below the age of 60 [113].

Measuring HDL function provides critical insights beyond total HDL-C levels alone across life stages including children, pregnancy, and the elderly. Like the elderly, key metrics of HDL function such as CEC appear to be impaired in other life stages, negatively impacting health. However, lifestyle interventions may restore function (Table 1). Implementing interventions that improve HDL function safely over decades is a desirable prevention approach starting early in life. Assessing HDL function across the lifespan will elucidate its complex roles in healthy versus unhealthy aging. Taking a longitudinal perspective enables connecting impaired HDL function to roles in disease development and preserves function long-term.

### 4.3. Activity of HDL-Associated Proteins/Components (e.g., PON-1, PAF-AH) in Aging

HDL presents additional functions besides cargo transportation, including antioxidation, anti-inflammation, anti-proteolysis, and anti-platelet formation [18]. Components associated with HDL mediate these additional functions, and changes to these components have been shown over the course of aging.

Research focused on dynamics of the aging HDL proteome is sparse. For example, HDL composition was altered in elderly participants, showing increased abundance of acute phase proteins, such as serum amyloid A and complement C3, and decreased abundance of ApoE, compared to young healthy subjects [114]. Additional compositional changes occur in the elderly as well, including lowered phospholipid layer fluidity and reduction in the phosphatidylcholine/sphingomyelin ratio of HDLs, which can affect non-ABCA1-mediated cholesterol efflux [110]. Increased sphingomyelin concentrations were also observed in elderly, potentially explaining the decreased phosphatidylcholine/sphingomyelin ratio [114].

Compared to HDL from young subjects (24.70 ± 3.09 y), elderly (74.86 ± 13.18 y) HDL samples had reduced antioxidation capacity, as shown by shorter lag-phase in the production of conjugated dienes when incubated with copper ion [115]. The reduced antioxidative capacity was highly associated with a reduced enzymatic activity of PON1, an HDL-associated protein responsible for preventing lipid oxidation. [115]. This observation was also supported by other groups, which confirmed defective antioxidant properties and lower PON1 activity in elderly HDL [114,116], and higher uptake rate by macrophages in elderly HDL samples compared to HDL from healthy young subjects [114].

Thus, in addition to the loss of beneficial functions, including antioxidant capacity, HDL from elderly individuals have also been shown to have gain of deleterious function. For example, Park and Cho 2011 showed that HDL from elderly subjects (71 ± 4 y, *n* = 26) compared to that from young males (22 ± 2 y, *n* = 18), had increased glycation with ApoA1 multimerization and decreased HDL phospholipid content, which induced more severe cellular senescence, foam cell formation when incubated with human dermal fibroblasts and macrophage, and induced cholesterol influx into macrophages [117].

In addition to enzymes like PON1 which decrease in the elderly, some HDL-associated proteins demonstrate increased activity with aging. Platelet-activating factor acetylhydrolase (PAH-AH) possesses anti-atherogenic properties by preventing LDL oxidation [100]. Campo et al., 2008 showed octogenarians from Sicily (84.18 ± 3.6 y, *n* = 100) had higher HDL-associated PAH-AH activity compared to healthy younger adults (41.0 ± 10.6 y, *n* = 200). This increase in PAH-AH may help compensate for declines in beneficial functions like antioxidant capacity due to altered levels of enzymes like PON1. The changes in both directions observed for proteins like PON1 and PAH-AH underscore the compositional dynamics occurring in elderly HDL, which influence overall function. Further research into the aging HDL proteome is needed to fully elucidate these compositional shifts and impacts on function.

Beyond cholesterol transport, HDL possesses additional functions mediated by associated proteins which change with aging. Overall, aging disturbs the proteomic composition of HDL, resulting in both beneficial and detrimental impacts on function. Further probing the dynamics of the HDL proteome alongside function during aging can elucidate mechanisms linking HDL to healthy longevity versus disease. Identifying protein targets for interventions may help preserve optimal function across the lifespan.

## 5. Assays to Assess HDL Function and Their Technical Considerations

A wide variety of functional assays have been described to assess HDL function [118]. Importantly, none of these assays, whether cell-based or cell-free, are standardized and instead vary in a number of ways from lab to lab. For example, multiple cell lines have been used to measure HDL CEC, including rodent immortalized cell lines such as J774A.1 [65,106], RAW 264.7 [119] and Fu5AH [120] as well as human immortalized cell lines HepG2 [65] and THP-1 [121]. Each cell line requires optimized growth conditions (e.g., media, incubation time, seeding density) to reduce the coefficients of variation (CVs) and ensure technical replicability both within an experiment and across experiments. CEC can be measured directly in plasma [122], ApoB-depleted plasma [123], as well as isolated HDL [59,106], and these different approaches are not comparable since the HDL particles are being dosed in different ways and agents that could be contributing to the efflux may or may not be present depending on the sample type. Furthermore, CEC can be measured using BODIPY-labeled cholesterol and radiolabeled cholesterol. The total fractional efflux of cholesterol is 3-fold higher with BODIPY-labeled vs. radiolabeled cholesterol (30% vs. 10% efflux) [124], which has been attributed to differences in the relative contribution of different efflux pathways (ABCA1 vs. ABCG1 and scavenger receptor class B member 1) between these two approaches [120]. Additionally, use of cyclic adenosine 3′,5′-monophosphate to stimulate ABCA1-mediated efflux, and acyl-CoA:cholesterol acyltransferase inhibitor to prevent cellular cholesterol storage, while dependent on the research question [125,126], also influences CEC values and reproducibility across studies. Despite optimization attempts to measure CEC using BODIPY-cholesterol [127], and other efforts achieving intra-plate and inter-plate CVs below 5% and 7%, respectively, using isolated HDL and BODIPY-labeled cholesterol [106], the lack of standardization makes comparing CEC across studies difficult. Other assays of HDL function such as HDL LCAT activity have shown excellent reproducibility when optimized, demonstrating intra-assay and inter-assay CVs below 2% and 5%, respectively, with isolated HDL [106]. However, HDL particle structure, function, and composition are influenced by the isolation method (e.g., ultracentrifugation, fast protein liquid chromatography, immunoprecipitation, and ApoB-depleted plasma) [75]. Thus, careful optimization and standardization are required to achieve reliable CVs across assays, with the selection balancing scientific relevance and reproducibility.

## 6. Challenges in Evaluating the HDL Hypothesis

The “HDL hypothesis” holds that HDL particles are causally protective against CVD because high HDL-C and ApoA1 concentrations are associated with reduced risk of CVD [128]. However, several issues have emerged surrounding this relationship, as all of the following are true: (1) it is possible to have high HDL-C and a *higher* risk of mortality [129], (2) it is possible to have low HDL-C and a *lower* risk of CVD incidence [130], and (3) drugs aimed at increasing HDL-C have not consistently reduced CVD risk [131]. In the first case, the mechanisms behind very high HDL-C and increased mortality are not completely understood; however, one contributing factor for high HDL-C is chronic alcohol use, which has been shown to increase HDL-C [132,133]. In the second case, genetically low HDL-C was observed in carriers of the ApoA1 Milano gene, whose HDL were found to be super functional and whose clearance was increased [130]. In the case of pharmaceutical approaches to increase HDL-C, the history of cholesteryl ester transfer protein (CETP) inhibitors is a long and complicated one, with many examples of the flaws in the pharmaceutical development pipeline to blame (e.g., the first CETP inhibitor was rushed through to phase III trials despite evidence of increased blood pressure in phase II trials which were later shown to be off-target effects of a hastily developed drug [134,135]). Despite the early failures, the CETP inhibitor development pipeline is still alive, with a recent phase II study showing patients on statin therapy in combination with obicetrapib, a new, potent CETP inhibitor, had lower ApoB, lipoprotein(a), and non-HDL-C concentrations, with concomitantly increased HDL-C and ApoA1 concentrations after 8 weeks of treatment [136].

For unknown reasons the HDL field seems to be fraught with errors and contradictions, which have led to complete confusion about the role and biology of HDL, and surprising findings which appear to condemn the HDL hypothesis. In addition to the controversies already described in the previous paragraphs, another example of this is Mendelian randomization studies, which have suggested that a causal relationship between plasma HDL-C and myocardial infarction does not exist [137]. Mendelian randomization studies can be a powerful way to explore causal relationships between a biomarker and a disease endpoint; however, given that certain assumptions need to be met, sometimes it is simply not possible to perform a meaningful Mendelian randomization analysis. This is likely the case with HDL: *APOA1* (the gene responsible for the synthesis of the defining HDL apoprotein and the primary driver of HDL synthesis), and *ABCA1* (the gene transcribing the primary receptor for HDL-mediated cholesterol efflux, the primary known HDL function)*,* despite being the primary genetic factors influencing HDL-C concentrations, were excluded from these analyses due to their pleiotropic effects on LDL-C and triglycerides [137]. The inability to include the effects of these major HDL-related genes in Mendelian randomization studies undermines any conclusions that can be drawn about the causal role of HDL-C in CVD, much as excluding *APOB* and *LDLR* from a Mendelian randomization study to investigate the causality of LDL-C in CVD would be meaningless.

HDL quality and function have been proposed as alternative measures to HDL-C concentrations. For instance, HDL CEC has been demonstrated to have a greater ability to predict CVD risk compared to HDL-C across multiple cohorts [20,22,138]. However, major uncertainties around HDL functional assays persist specifically as it pertains to their clinical validity. The complexity of HDL functional assays and limited standardization restrict their utility for both therapeutic development and for evaluating the HDL hypothesis until thorough validation in long-term studies using hard clinical endpoints becomes available [139]. HDL functionality is also being investigated as a primary endpoint for HDL-based therapeutics. An ongoing Phase-III clinical trial for CSL-112, a reconstituted HDL particle consisting of ApoA1 purified from human plasma combined with phosphotidylcholine, has shown promise to potentially reduce CVD risk by remodeling endogenous HDL to improve CEC and LCAT activity [140,141], both of which are decreased in myocardial infarction patients [142,143].

Further complicating research in the HDL field is that any measure of HDL function is influenced by total particle concentration as well as the size distribution of the particles [144], which can be highly varied among individuals. For example, when assessing CEC of isolated HDL particles, HDL is typically dosed by protein content, which can be confounded by differences in HDL subclass distribution [106]. Since small HDL particles have a higher percentage of protein compared to large HDL (56% vs. 40% by weight) [145], evaluating the CEC of an equal dose of HDL as µg of protein could potentially confound the results because, in effect, the sample with a higher proportion of small HDL would have higher numbers of particles in the well for the same protein dose. Dosing by any other constituent (e.g., ApoA1, phospholipid, lipid) has the same problem, as different sized HDL particles have different relative and absolute contents of all of these constituents. While HDL particle number has shown a stronger association with CVD risk compared with both HDL-C and CEC [22], it remains unclear whether increasing the total number of HDL particles, increasing a specific subset of HDL particles (e.g., small HDL), or increasing the functionality of all or a subset of particles, is the correct intervention target. Importantly, although CVD is the most well-researched disease context for HDL particles, emerging evidence, as discussed throughout this paper, points to the need for further study of the relationships between HDL particle concentration, size distribution, composition and function in a wider variety of population groups, including pregnant women, children, and the elderly.

## 7. Remaining Gaps and Future Research Directions

Major uncertainties persist around the validity of HDL functional assays, as detailed above. Additionally, improved HDL function resulting from an intervention does not definitively establish a causal role of that function in mediating clinical outcomes. Many interventions likely act through multiple pathways beyond HDL, underscoring the need to isolate the specific effects of improved HDL function. For example, CETP inhibition raises HDL while also lowering LDL cholesterol [136]; thus, cardiovascular benefits may relate more to reduced LDL-C rather than increased HDL-C. Further research is needed to conclusively link health outcomes to enhanced HDL function itself. Knowledge gaps also remain in understanding HDL biology across the lifespan. There is a need for longitudinal studies tracking HDL starting in early life to elucidate developmental trajectories into adulthood. Dynamic changes to HDL subclasses during pregnancy and their impacts on maternal and child health are poorly characterized. Research elucidating the shifts in HDL function, composition and subclass distribution in the elderly is lacking. Multidimensional comparisons of HDL parameters across life stages are needed to delineate age-related HDL changes from dysfunctional HDL in disease states.

To address these gaps, future HDL research should utilize advanced isolation techniques, functional assays, omics analyses and longitudinal study designs. Exploring interventions that optimize HDL function at all life stages may uncover novel prevention approaches. Filling remaining knowledge voids will provide insights into HDL’s complex roles in health versus disease and open new avenues for diagnostics and therapies targeting HDL to improve outcomes across the lifespan.

## 8. Conclusions

**Figure 1:**
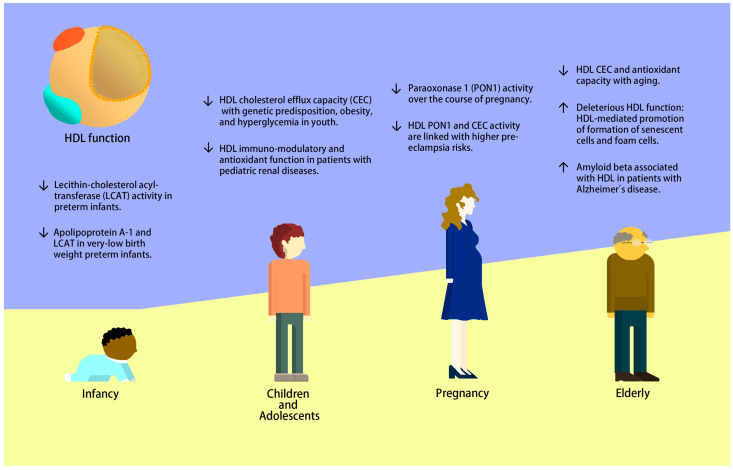
HDL changes associated with disease risk across the lifespan.

HDL has the potential to be a predictive measurement of health status across all life stages, from infancy, through pregnancy, and in aging (Figure 1). Similar to adults, children with underlying conditions, such as dyslipidemia, diabetes, and chronic kidney disease, exhibit some degree of HDL dysfunctionality, but many gaps in knowledge remain and much further work is needed to understand the role of HDL in children on health status later in life. HDL-C and HDL CEC are established predictors of CVD risk, but HDL-C does not reflect HDL function. During pregnancy, the time at which HDL is measured is essential. Changes to HDL subclasses occur throughout gestation, but it is uncertain how these alterations relate to HDL function at different stages of pregnancy, and how these alterations affect pregnancy outcomes and life-long trajectories in the infant. In certain conditions such as metabolic syndrome where the underlying mechanism of loss of function is the loss of total numbers of HDL particles, the measurement of HDL-C, total HDL particle count, and total ApoA1 in plasma may be adequate. However, in other physiological states such as pregnancy, simple measurements of total amounts of HDL in circulation do not appear to be useful because it is instead the loss of certain subsets of HDL particles or certain functional aspects of HDL that have negative health effects. In elderly individuals, HDL function and composition change as part of the aging process; however, this is highly differential between healthy elderly individuals achieving very long lifespans, such as octogenarians, nonagenarians, and centenarians, compared with elderly individuals with chronic diseases such as CVD and AD. The study of healthy vs. unhealthy elderly individuals will be an important area of research for the discovery of protective mechanisms that improve health outcomes, as well as loss-of-beneficial-function and gain-of-deleterious-function aspects of HDL. Most work on HDL in elderly, as well as in children and in pregnancy, is cross-sectional, hindering our ability to derive casual relationships. Thus, in addition to further studies on the functional biology of HDL at these under-studied life stages, future work is needed to fully understand how different conditions and physiological states affect the components of HDL, including function, subclass distribution, and composition. HDL particles have multi-faceted links with health status across life stages, with early influences as early as in utero and in infancy having lasting life-long effects. As the HDL field continues to advance and as more sophisticated methods for isolating and characterizing HDL emerge, new discoveries will lead to novel HDL-based diagnostics and interventions/therapeutics. Interventions to modify and improve the functionality of HDL particles can improve health outcomes and health trajectories at all life stages.

## Figures and Tables

**Table 1:** Interventions associated with improved HDL cholesterol levels and function across different age groups.

Study	Intervention	Outcome	Reference
Singhal et al., 2004	Preterm infants given breast milk early in life vs. preterm formula.	Decreased both LDL:HDL and ApoB:ApoA1 ratios at adolescents.	[25]
Harit et al., 2008	Full term infants given breast milk exclusively for 6 months vs. mixed feeding.	Increased infant serum HDL-C:LDL-C ratios.	[26]
Genzel-Boroviczény et al., 2002	Early total parenteral nutrition in preterm infants (≤32 weeks gestational age) from birth to 5 days of age.	Increased infant plasma ApoA1.	[30]
Hong et al., 2021	Maternal (2nd trimester to birth) and their children (0–6 months) supplemented with lipid-based nutrient supplements.	Increased children HDL cholesterol efflux capacity (CEC) at 18 months of age.	[59]
Davidson et al., 2017	Vertical sleeve gastrectomy in obese adolescent males.	Increased both HDL CEC and anti-oxidative activity 1-year post-operative.	[62]
Wesnigk et al., 2016	10-month lifestyle intervention of aerobic activity and nutrition counseling in obese adolescents.	Increased both HDL-C and HDL CEC.	[64]
Otrante et al., 2021	12-weeks consumption of extra virgin olive oil (25 mL/day) in elderly.	Increased HDL CEC.	[111]

## Data Availability

Not applicable.
